# Biomechanical effects of digitally constructed titanium, modified polyetheretherketone, and polyetherketoneketone subperiosteal implants on atrophied maxilla: a finite element analysis

**DOI:** 10.1186/s12903-025-06426-z

**Published:** 2025-07-10

**Authors:** Mohammed A. El-Sawy, Basin El-Khatib, Hesham S. Borg, Mohamed T. Khater

**Affiliations:** 1 Department of Prosthetic Dental Science, Faculty of Dentistry, Menoufia University, Shibin El-Kom, Egypt; 2 Department of Prosthetic Dental Science, Faculty of Dentistry, National Menoufia University, Tukh Tanbisha, 6131567 Menoufia, Egypt; 3 Ministry of Health and Population, Hospital st., Gharbia 6783504 Samanoud, Egypt; 4 Assistant Professor of Prosthodontics, Faculty of Dentistry, Alsalam University, Abyar, Kafr El-Zayat , 42525 Gharbia, Egypt; 5 Department of Oral and Maxillofacial Surgery, Faculty of Dentistry, Menoufia University, Shibin El-Kom, Egypt

**Keywords:** Finite element analysis, Mini screw, Subperiosteal and superstructure frameworks, Maxillary fixed prosthesis, Titanium, Modified PEEK, PEKK

## Abstract

**Aim:**

This study aimed to evaluate how different combinations of subperiosteal and superstructure framework materials—titanium, modified PEEK, and PEKK—affect stress distribution on bone in atrophic maxillae, using finite element analysis (FEA).

**Methods:**

A three-dimensional finite element model of an atrophic maxilla was created from CT data and processed through CAD and ANSYS software. Nine combinations of framework materials were tested under three loading protocols (vertical, oblique, and incisor-directed forces). The subperiosteal framework was fixed in place by 12 mini-screw with different lengths, that the 3 materials were assigned to frameworks in 9 different combinations for the lowest stresses on bone. Three different loading protocols were applied to the prosthetic structure with each of frameworks materials combination.

**Results:**

Titanium subperiosteal frameworks transferred the least stress to underlying bone and fixation screws, while modified PEEK and PEKK showed higher stress values, particularly under incisor loading. Titanium superstructures exhibited higher internal stresses due to rigidity but protected supporting structures more effectively. Cases under vertical incisors forces showed very high stress levels on cement layer and subperiosteal frame due to bending, and high stresses on mini-screws and bone. These levels were critical for cement layer, modified PEEK, and PEKK framework materials, bone, except, mini screws, that stresses level were in the safe region.

**Conclusion:**

Within the limitations of this FEA analysis, Titanium is the optimal material for subperiosteal frameworks in atrophic maxillae due to its superior stress distribution. PEKK and modified PEEK may be viable alternatives in patients with reduced functional loading.

**Clinical significance:**

Titanium subperiosteal framework should be considered the optimum material for subperiosteal implants. Modified PEEK and PEKK material can be considered as alternative material to titanium subperiosteal framework for patients with lower masticatory forces (complete denture on the opposite arch or on the anterior segment).

**Clinical trial registry number:**

Registered at www.clinicaltrials.gov (NCT06362057) (2024-04-8).

## Introduction

Atrophied jaw is a condition in which there is insufficient bone to support an endosseous (root-form) implant. Patients with atrophic jaws have a variety of therapeutic options, including sinus lift, guided bone regeneration, pterygoid implants, zygomatic implants, inferior alveolar nerve lateralization, and distraction osteogenesis.

A patient may reject the treatment plan for a fixed prosthesis with the previous options due to its disadvantages, which include, time-consuming surgery, invasiveness, the need for second stage surgery, high morbidity and long intervals between the initiation of therapy and the delivery of the definitive superstructure [1–3]. Rehabilitation after a single-stage procedure is readily acceptable for individuals with severely resorbed edentulous arches.

Instead of being placed directly in the jawbone, subperiosteal implants are placed under the gingiva and on top of the bone with transmucosal abutments emerged from the gingiva. The subperiosteal jaw implant is not a novel concept. The first subperiosteal frames were conceived by Dahl in the 1940s, It later lost favor when root-form implants emerged as the gold standard for permanent dental prosthesis by the 1980s. However, root-form implants are not suitable for all patients.

Advances in radiographic method, bone segmentation software’s, dental materials, and digital technology of fabrication such as milling and three dimensional (3D) printing have prompted a reconsideration of previous concepts, including subperiosteal implants [4, 5], This made it feasible to treat not just atrophied jaws but also medically compromised patients and massive maxillary defects (maxillectomies) [6–12].

The value of the subperiosteal implant has decreased due to the advancements in root-form implants during the past 15 years [13, 14]. But since root-form implants aren’t suitable for every patient, a lot of new research is being done on the new generations of subperiosteal implants [15–17].

Strength, biocompatibility, corrosion resistance, toughness, fracture, and wear resistance are all essential characteristics of an ideal implant biomaterial [18, 19]. Titanium and its alloys, particularly Ti6Al4V (Ti grade 5), are widely used in implantology due to their excellent mechanical properties and proven clinical success [20]. However, their high stiffness compared to bone may lead to stress shielding, potentially contributing to bone resorption over time [21].

To address these limitations, high-performance polymers such as polyetheretherketone (PEEK) and polyetherketoneketone (PEKK)—both members of the poly(aryl-ether-ketone) (PAEK) family—have emerged as promising alternatives [22, 23]. These materials have a modulus of elasticity closer to that of cortical bone, which may reduce stress shielding and improve load transfer to the surrounding bone. Their favorable biomechanical properties, along with chemical stability and biocompatibility, make them particularly suitable for patients with compromised bone quality or density, and for those in whom conventional root-form implants are contraindicated due to systemic health conditions or anatomical limitations.

BioHPP, a modified version of PEEK reinforced with 20% ceramic fillers, offers enhanced strength, wear resistance, and veneering capability, making it especially attractive for prosthetic superstructures [23]. PEKK, due to its higher compressive strength and thermal stability compared to PEEK, is considered a next-generation polymer for load-bearing applications [23]. Evaluating these materials in both subperiosteal and superstructure roles may provide insight into optimized solutions for high-risk or medically complex patients.

Stress analysis using FEA has become essential in prosthodontics and implantology for evaluating the mechanical behavior of different materials [20, 24, 25]. Previous FEA studies have evaluated isolated frameworks [20, 25, 26], but none have examined the combined effect of subperiosteal and superstructure material choices. Therefore, the unique contribution of this study is to fill this gap using FEA to assess the effect of different material combinations—titanium, modified PEEK, and PEKK—on stress distribution in the bone under masticatory forces. The null hypothesis was that the bone would respond similarly regardless of the materials used for the subperiosteal implant and superstructure.

## Materials and methods

### Model construction

**Fig. 1:**
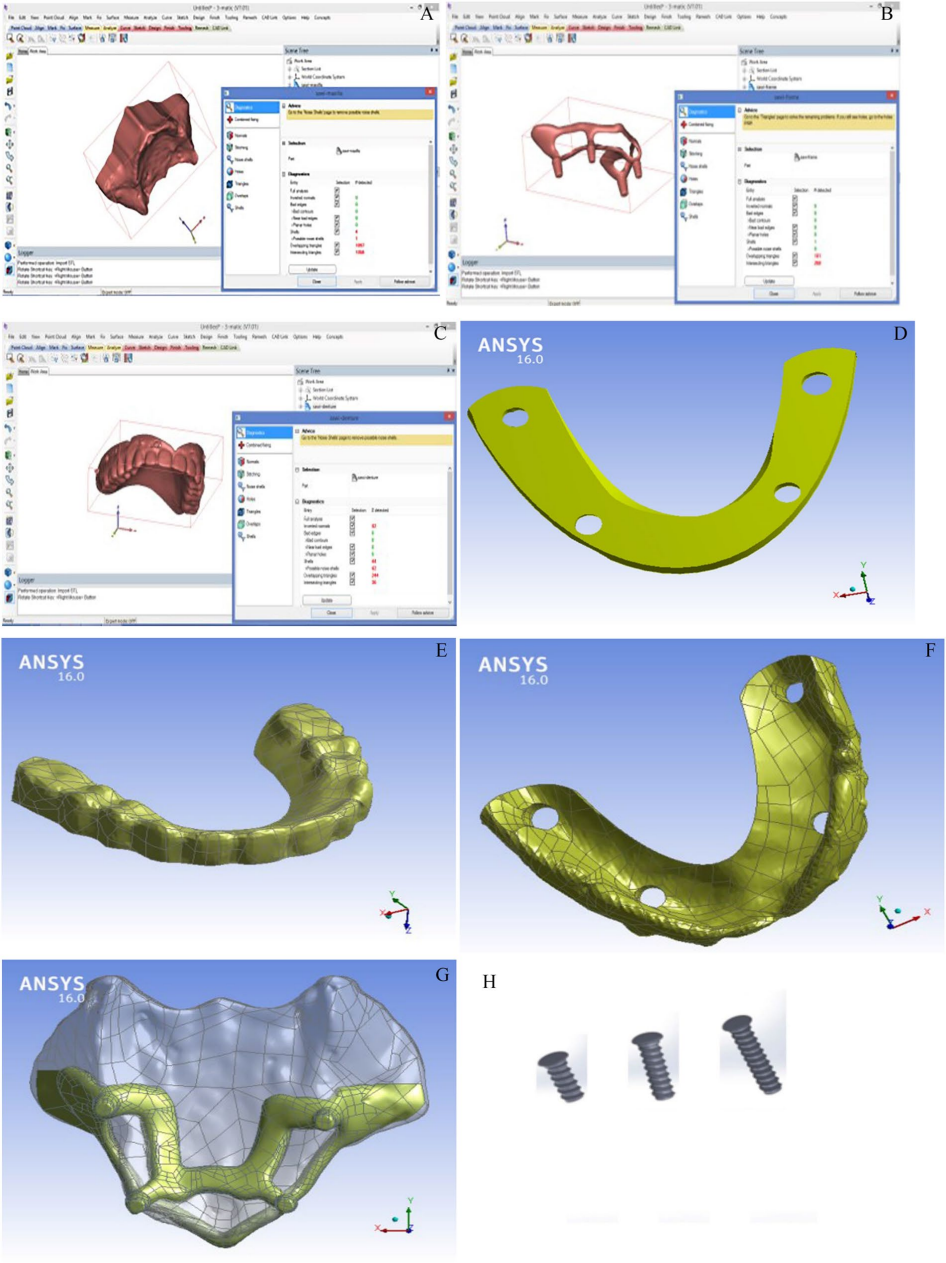
**A**, **B**, and **C** are scanned parts on the 3Matic screen showing STL files. **D**, views of the superstructure framework. **E**, views of the superstructure teeth top view. **F**, views of the superstructure teeth bottom view. **H**, Views of the subperiosteal framework. **I**, used mini screws of 2 mm diameter and lengths of 5, 7, and 9 mm to fix the subperiosteal framework on the bone

To give a more precise description of a person’s specific state, the geometric models for maxilla, subperiosteal framework, and superstructure were obtained via computed tomography (CT) images derived from a previous study (Fig. 1). These images passed through standard methodology steps to create cloud of points for each of the models’ component, which is known as stereolithography (STL) file. STL file was handled on computer-aided design (CAD) software. This format approximates the surfaces of a solid model with triangles. Therefore, intermediate software was utilized (3Matic; Materialise, NV) to trim the newly created surfaces by the acquired points. Then, the solid (closed) component geometry was finally checked by SolidWorks 2014 (13090 Aix-en-Provence, Dassault Systèmes Inc.) prior to be exported to finite element program as STEP file format [27]. The FEM was validated by comparing the mode shapes and applying similar loads (both in magnitude and location) as described in the referenced studies [5, 20, 26]. The resulting stress distributions and values were then compared, revealing very good agreement.

The subperiosteal framework was fixed in place by 12 mini-screw (Ø2-mmdiameter) with lengths of 5-, 7-, and 9-mm lengths which were created by commercial CAD software SolidWorks 2014 (13090 Aix-en-Provence, Dassault Systèmes Inc.) As recommended by Dimitriou et al., [17] a minimum of 8 screws buccally and 2 or more palatally is advised to ensure adequate primary stability and effective distribution of functional loads across the baseplate’s surface. This configuration enhances the mechanical performance and helps in minimizing stress concentrations. Furthermore, screws were positioned in regions of the jaw that offer the highest anatomical resistance, in line with biomechanical principles. The variation in screw lengths (5, 7, and 9 mm) was employed to accommodate anatomical constraints and avoid interference with critical anatomical structures such as nerves, and sinus cavities.

### Material properties

Three materials were assigned to both subperiosteal framework and prosthesis superstructure in 9 different combinations for the lowest stresses on bone. The amount of cement space between the subperiosteal abutment and the prosthesis framework was about 110 microns.

This value was chosen based on various studies [28, 29], indicating that modifying the cement spacer to 110 μm improves the marginal adaptability of CAD/CAM crowns without compromising their retention. Material assignment is one of the preprocessing steps in the simulation process, where a specific volume (part of the geometric model) is selected and assigned material properties such as the modulus of elasticity and Poisson’s ratio (Table 1).

**Table 1:** Material properties of used in the finite element model(s)

Material	Tradename / Manufacturer	Modules of Elasticity [MPa]	Posison’s ratio	Reference
Prosthesis Superstructure “Teeth” *(Acrylic veneers)* Polymethylmethacrylate (PMMA) veneers	Novo. lign, Bredent GmbH & Co. KG, Senden, Germany	3,000	0.35	^[26]^
Pink “Gingiva” *(pink acrylic resin)*	Crea. lign, Bredent GmbH & Co. KG, Senden, Germany	2,700	0.35	^[48]^
Cement layers	(DTK-Kleber adhesive, Bredent, Germany)	8,000	0.30	^[49]^ ^[50]^
Framework material 1: Titanium	Ti-6Al-4 V	111,000	0.34	^[20]^
Framework material 2: Modified PEEK	Bio- HPP; bredent GmbH & Co KG	4,000 (4,200-4,800)	0.37	^[51]^ ^[52]^
Framework material 3: PEKK	Pekkton Ivory; Cendres + Métaux SA	5,100	0.40	^[44]^
Mini-Screws	Ti-6Al-4 V	111,000	0.34	^[20]^
Bone	-	15,000	0.30	^[20]^

### Meshing and boundary conditions

The next step is meshing, in which each part of the model is divided into very small elements with defined geometries and mechanical properties within the domain.

Finally, a set of Boolean operations on ANSYS environment (Canonsburg; ANSYS Inc) that, were performed to finalize the tested model. On the other hand, all materials fed to ANSYS were considered isotropic, liner and elastic.

**Fig. 2:**
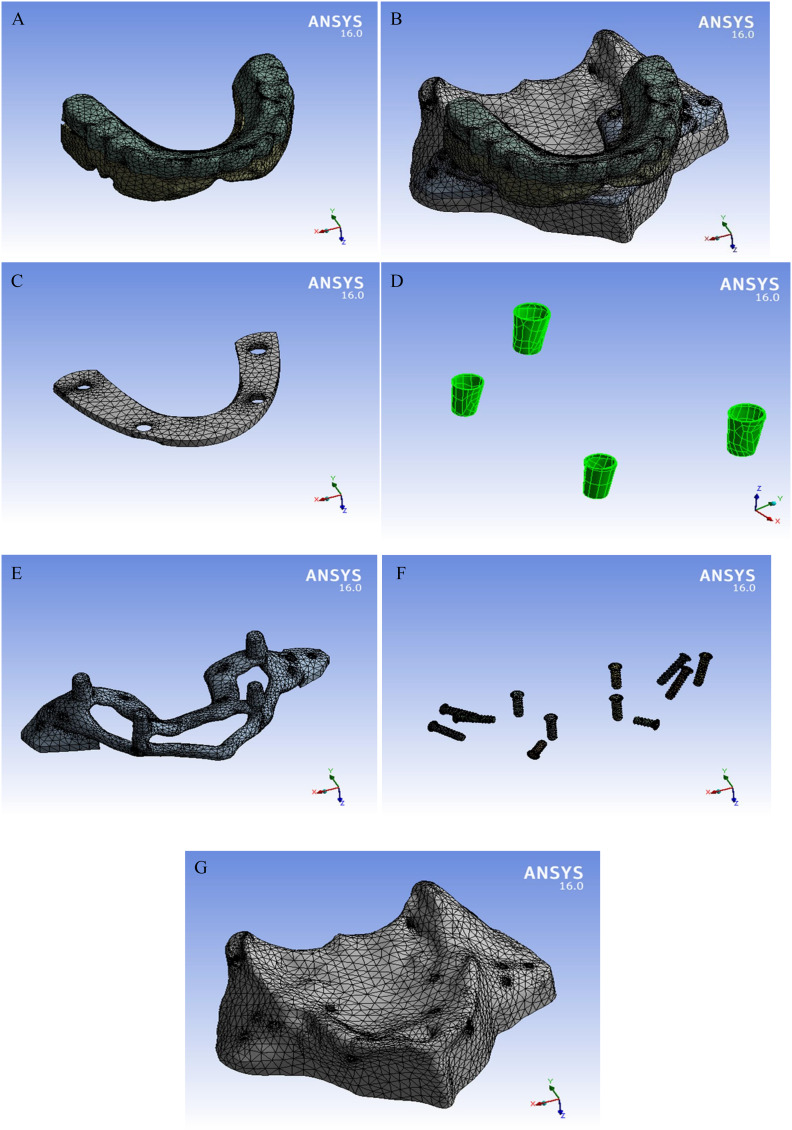
Screen shots for model components and their mesh. **A**, full model. **B**, overdenture. **C** superstructure framework. **D**, cement layer. **E**, subperiosteal framework. **F**, twelve mini-screws. **G**, cortical bone

The meshing of the models’ components was created by 3D brick solid element “187” which has 3 degrees of freedom (translation in main axes directions) [30]. Meshing convergence test (less than 3%) was performed to ensure results accuracy. The resulted numbers of nodes and elements are listed in Table 2 and the meshed model components are presented as screenshots from ANSYS (Fig. 2).

**Table 2:** Mesh density of the two models’ components

Part / Material	Number of elements	Number of nodes
Superstructure (Teeth & Gingiva)	146,345 111,153	102,392 78,194
Superstructure Framework	40,210	27,252
Mini screws (12 screws)	82,812	51,420
Cement	56,535	28,137
Subperiosteal Framework	190,928	130,529
Bone	281,768	201,233

### Evaluation of loading protocols

Three different loading protocols were applied to the prosthetic structures for each framework material combination: (1) Vertical posterior loading: A bilateral vertical force of 200 N was applied to the first premolars, second premolars, and first molars reciprocally. This load magnitude reflects the typical range of maximum posterior bite forces encountered during mastication, which may reach 200–600 N in healthy adults, depending on individual variation and tooth location [31, 32]. (2) Oblique posterior loading: A bilateral 100 N oblique force inclined at 30° was applied to the same posterior teeth. This condition simulates functional masticatory forces under non-axial chewing, such as during lateral excursions or dynamic occlusion, where oblique force vectors are lower than pure vertical loads [33, 34]. (3) Anterior loading: A total vertical force of 150 N was distributed across the central and lateral incisors. This represents physiological anterior bite forces, which are typically lower than posterior forces but may reach up to 150 N during activities like incising or clenching [35].

**Fig. 3:**
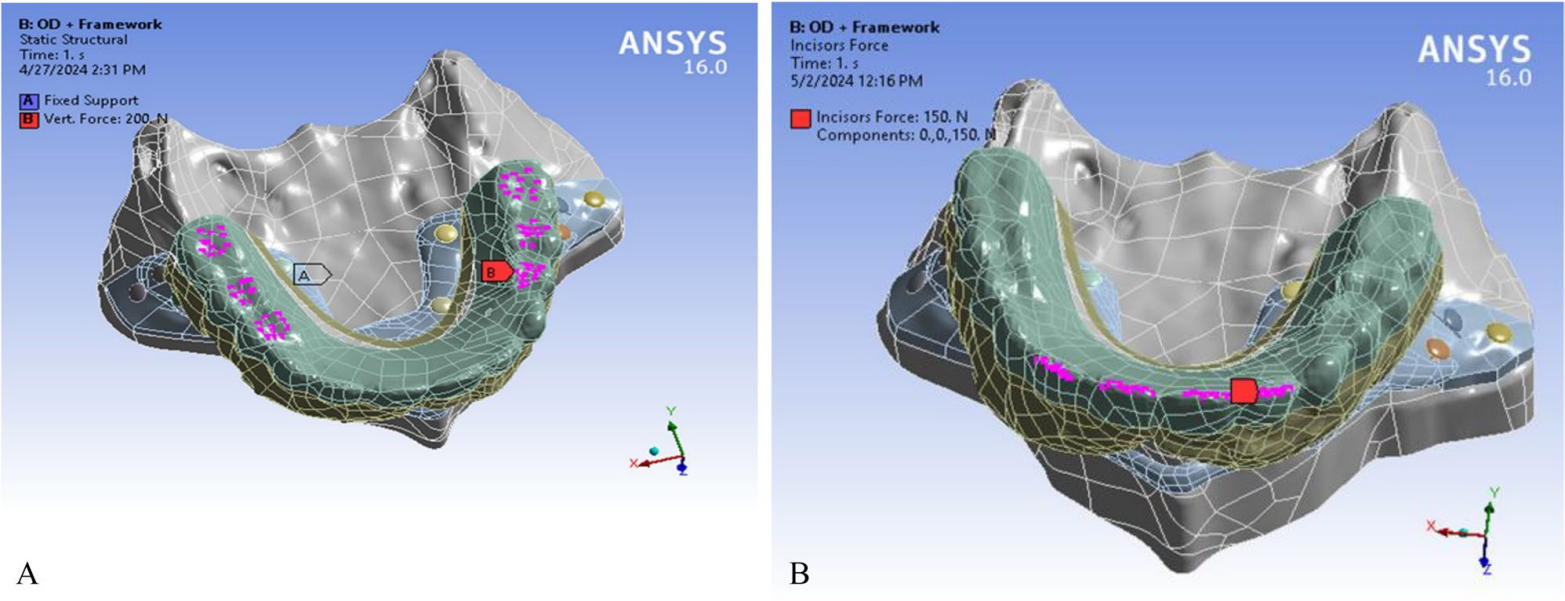
Loading location in the three protocols

These force magnitudes were selected to reflect clinically relevant loading scenarios in both static and dynamic occlusion, enabling realistic simulation of stress distribution patterns within the prosthetic framework and surrounding bone. (Fig. 3)

**Fig. 4:**
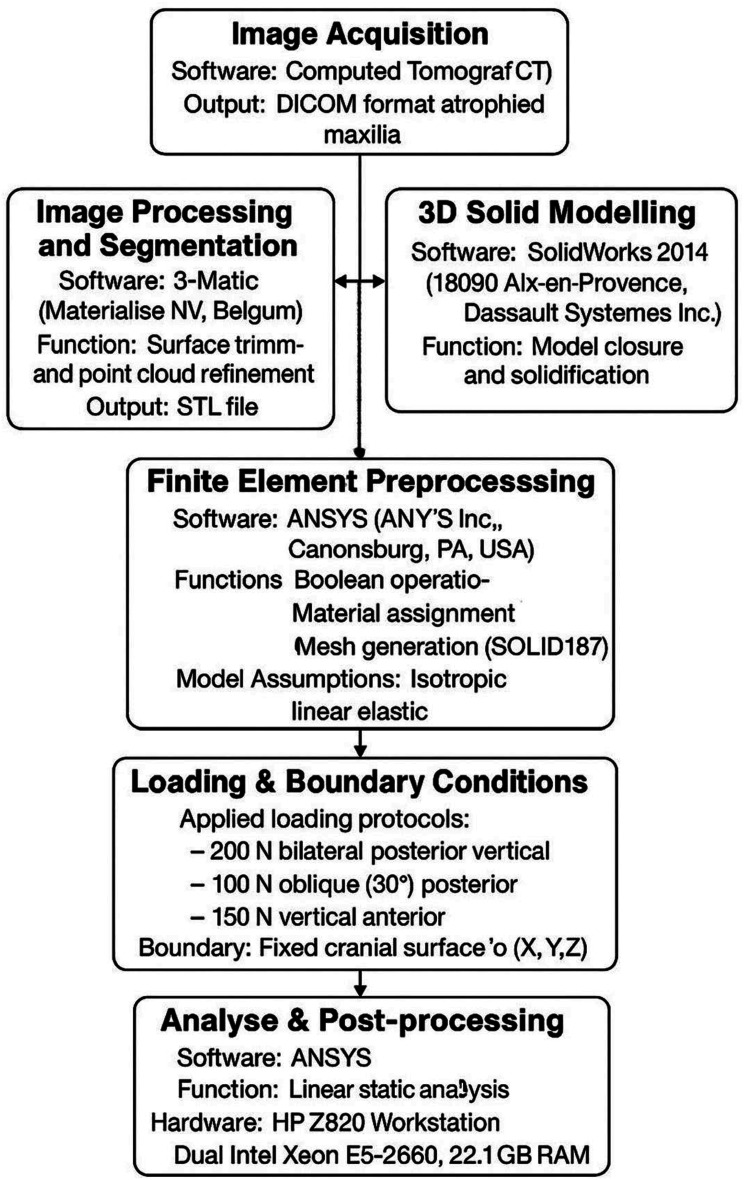
Flow diagram outlining the sequential use of software tools in the modeling and analysis process

The model was verified against similar studies [5, 20, 26] prior to extracting the analysis results. As a boundary condition, the superior surface of the maxilla (cranial side) was fixed in all three directions (X, Y, and Z). This constraint mimics the natural, immobile connection of the maxilla to the skull base and adjacent bones, accurately reflecting the real-world mechanical boundary conditions of the maxilla within the craniofacial skeleton. Linear static analyses were performed on a Workstation HP Z820, with dual Intel Xeon E5-2660, 2.2 GHz processors, and 64 GB RAM. Figure. 4, outlines the sequential use of software tools in the modeling and analysis process.

## Results

A lot of graphical distributions for deformations and stresses resulted from analyses. Comparisons between the 27 case studies results on each component under the same loading condition can led to conclusions. Deformation comparisons took the same trends for each component under the same loading protocol but with variation in values. Quantitative comparisons in this study were based on von Mises stress values, principal stresses, and displacement magnitudes, with percentage differences and stress ratios included where relevant to support material and design comparisons. Titanium subperiosteal frame gives the lowest values of deformation. While super structure framework material keeps the trend as titanium, PEKK, and modified PEEK as increasing values. Across all loading conditions (vertical posterior, oblique posterior, and incisor-directed), distinct numerical trends emerged in stress and deformation behavior depending on the material combinations.

Stresses showed different trends according to the component and the applied load protocol. Therefore, the superstructure (teeth & gingival) did not show any significant change in stress values or distribution with changing framework materials. Regardless of the material used, stress values on the superstructure ranged from 35 MPa under vertical loading to about 60 MPa under incisor loading.

**Fig. 5:**
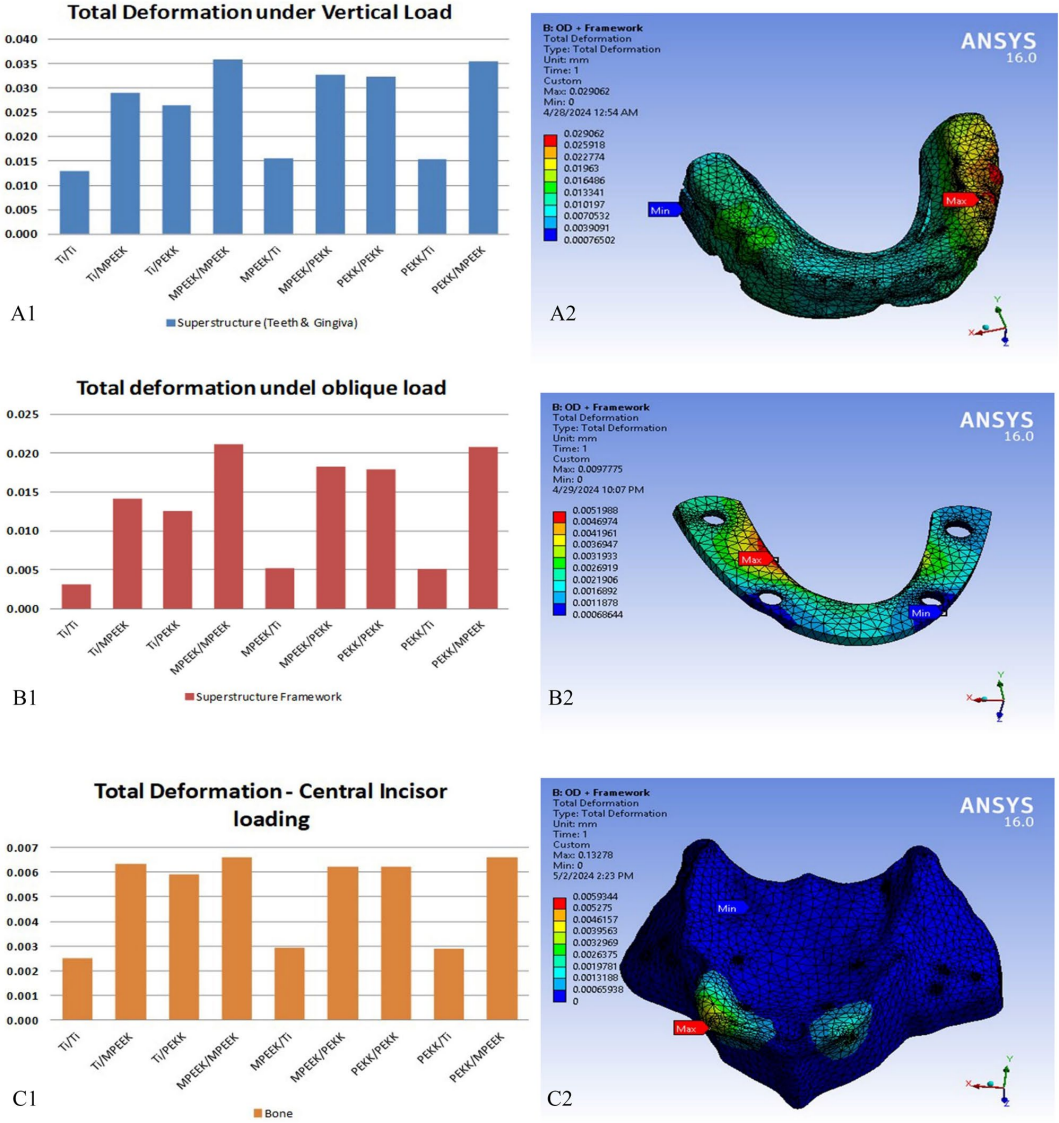
Sample of total deformation results and its comparisons. **A**, superstructure under vertical loading; **B**, superstructure framework under oblique loading. **C**, bone under incisors vertical loading

Bone stresses were consistently lowest with titanium subperiosteal frameworks. Under vertical loading, maximum principal bone stress was ~ 16 MPa with titanium, compared to 28 MPa with modified PEEK. Under oblique loading, the range was 15 MPa (titanium) to 26 MPa (modified PEEK). Under incisor vertical loading, stress peaked at ~ 65 MPa for modified PEEK, 58 MPa for PEKK, and 39 MPa for titanium, the only material maintaining values below physiological limits. Figure 5 showed samples of total deformation results comparisons on superstructure, superstructure framework, and bone under vertical, oblique, and incisors vertical loading respectively.

Cement layer stress followed a similar trend. Under vertical and oblique loading, titanium frameworks resulted in the lowest cement stress (~ 20 MPa and 18 MPa, respectively), while modified PEEK peaked at 35 MPa (vertical) and 28 MPa (oblique). Under incisor loading, cement stresses reached 50 MPa with modified PEEK and 35 MPa with titanium, nearing failure thresholds.

**Fig. 6:**
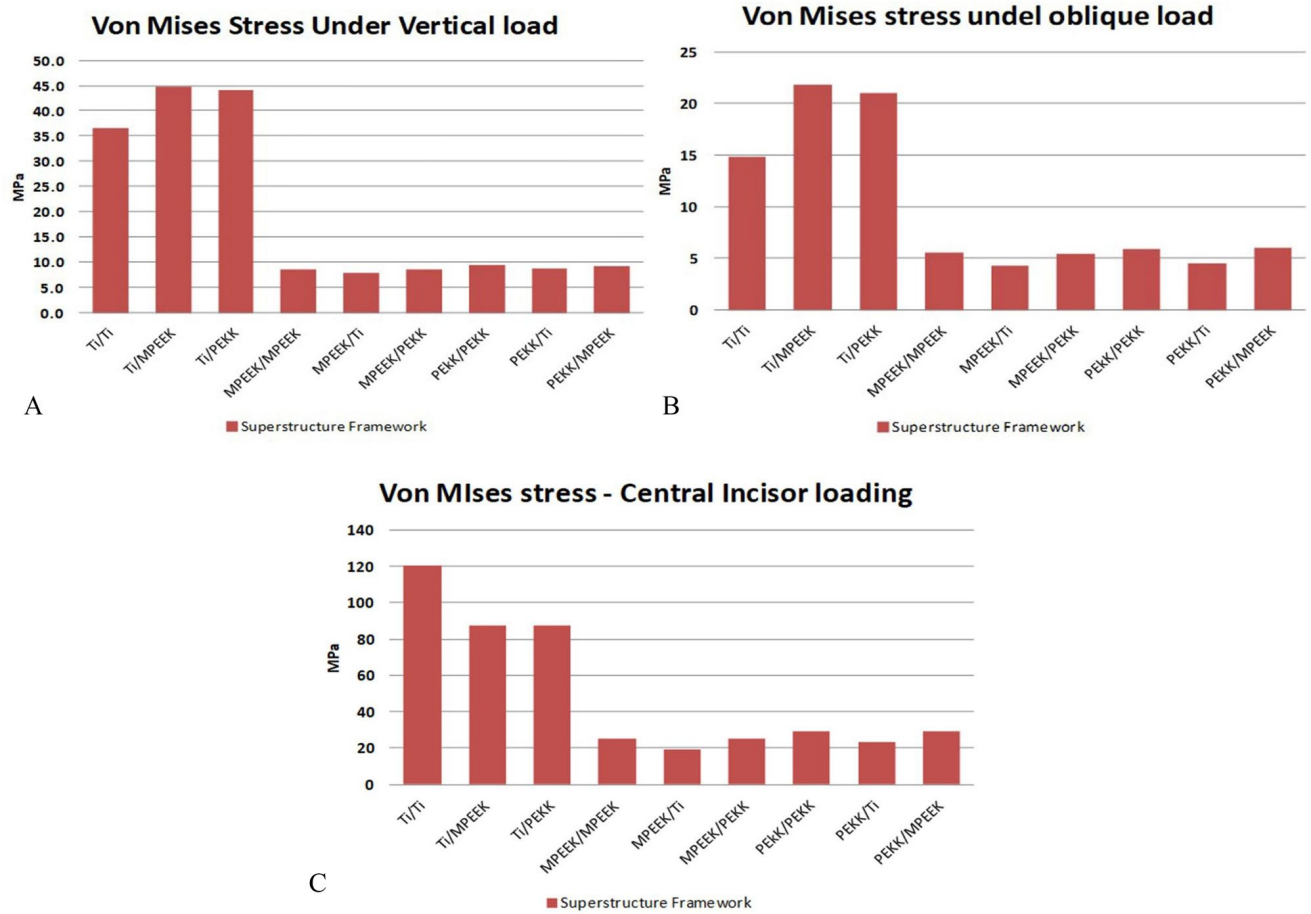
Von Mises stress value comparisons on the superstructure framework under different loading protocols. **A**, bilateral vertical loading. **B**, bilateral oblique loading. **C**, incisors vertical loading

**Fig. 7:**
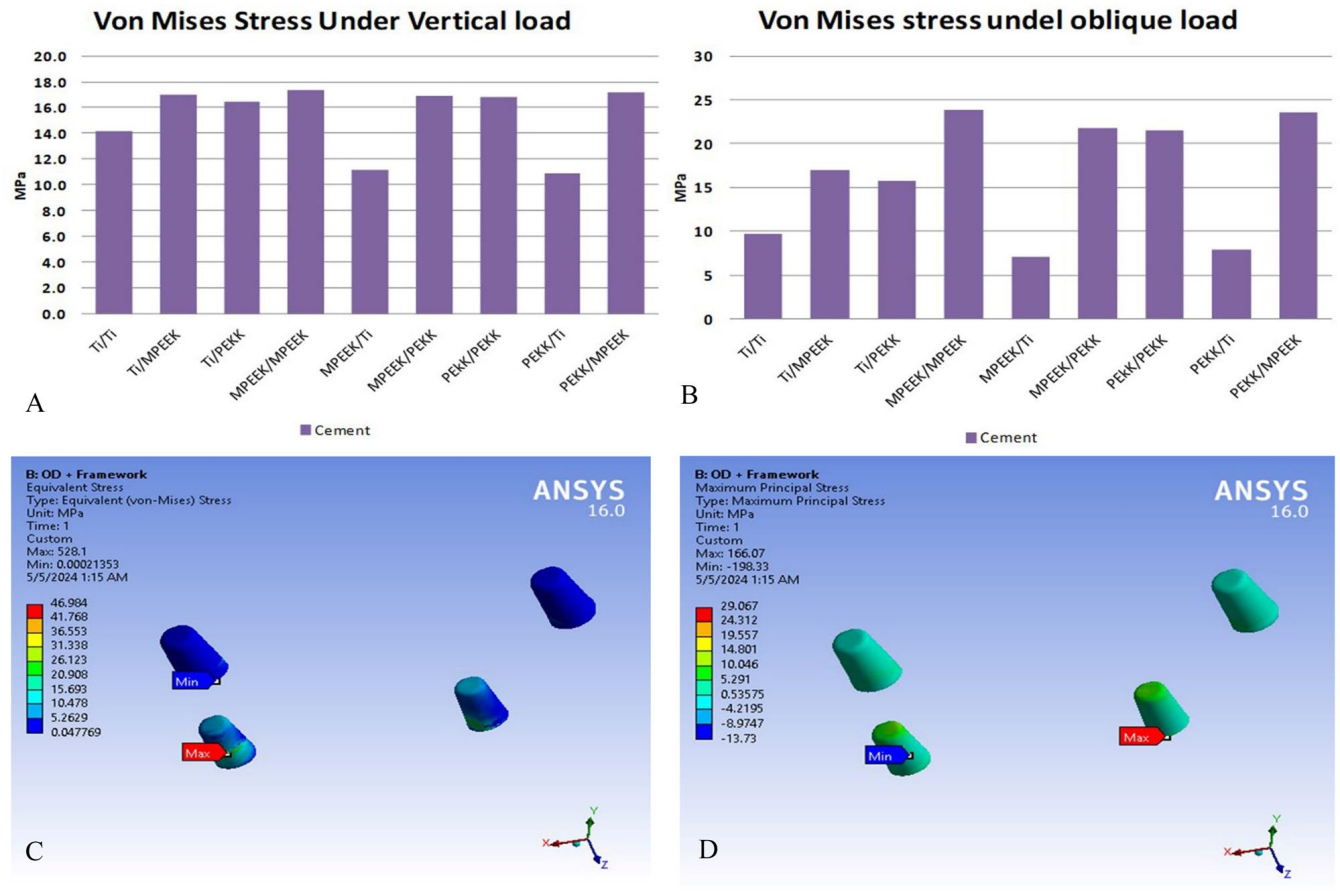
Comparison of stresses: extreme values appeared on the cement layer and samples of its stress distributions with PEEK/PEEK frameworks under incisor vertical loading. PEEK, Polyetheretherketone

Superstructure framework stress was highest when titanium was used due to its stiffness. Under vertical loading, titanium reached ~ 130 MPa, compared to 45 MPa (PEKK) and 40 MPa (modified PEEK). Under oblique loading, the titanium framework recorded ~ 150 MPa, while PEKK and modified PEEK were around 50 MPa and 45 MPa, respectively. Under incisor loading, titanium again peaked at 160 MPa, while PEKK and modified PEEK remained under 50 MPa. All values were within material safety limits. (Figures 6 and 7).

**Fig. 8:**
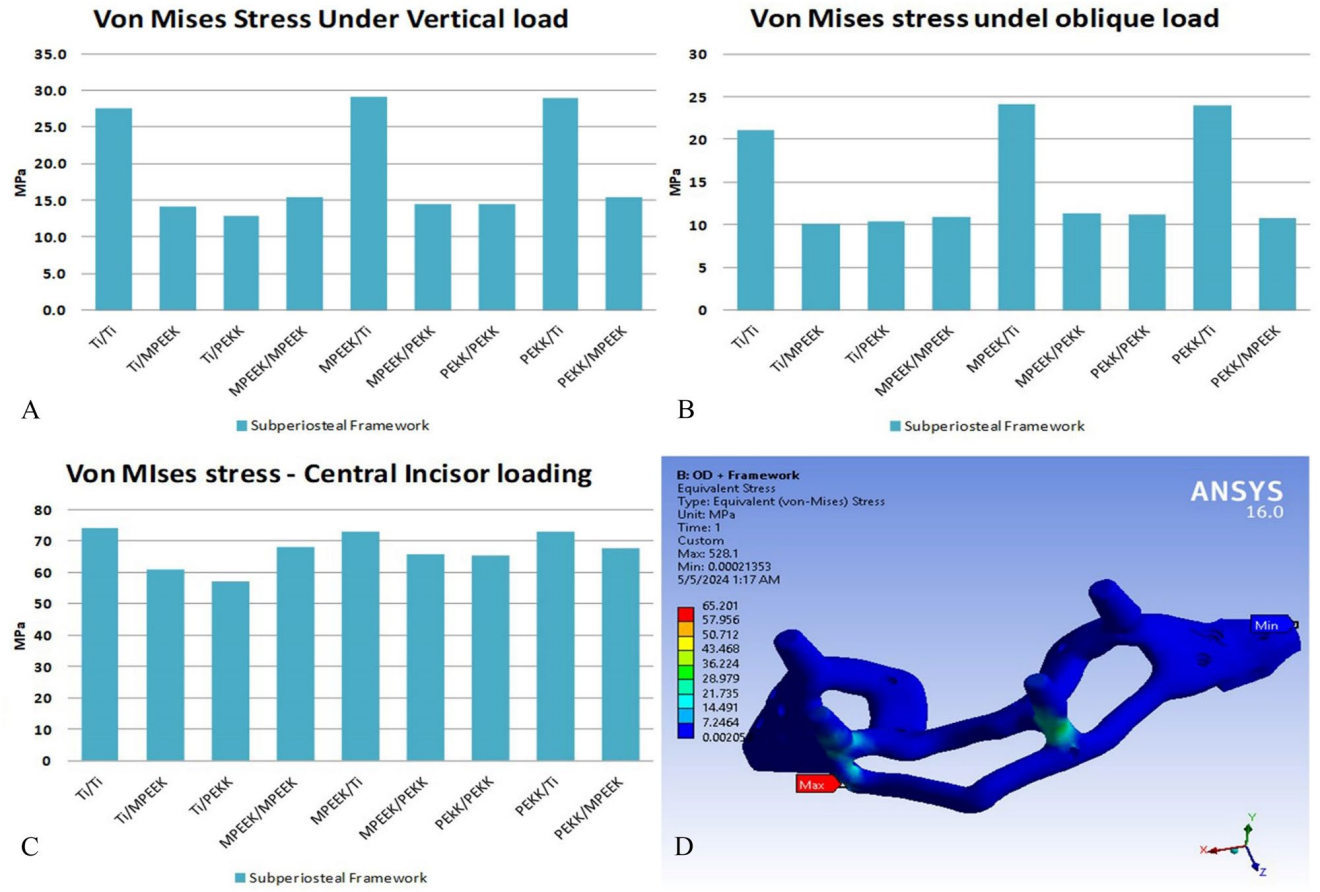
Comparison of stresses: extreme values appeared on the subperiosteal frame and sample of its stress distributions under incisor vertical loading

Subperiosteal framework stress showed material-dependent behavior. Under vertical loading, titanium frameworks bore internal stresses up to 110 MPa, compared to 60 MPa (PEKK) and 55 MPa (modified PEEK). Oblique loading showed a similar trend. Under incisor loading, all materials showed 60–70 MPa bending stress. While safe for titanium, these values approached the yield strength of modified PEEK and PEKK, particularly when combined with more flexible superstructures, potentially limiting their clinical longevity to ~ 10,000 cycles (approx. one year) (Fig. 8).

**Fig. 9:**
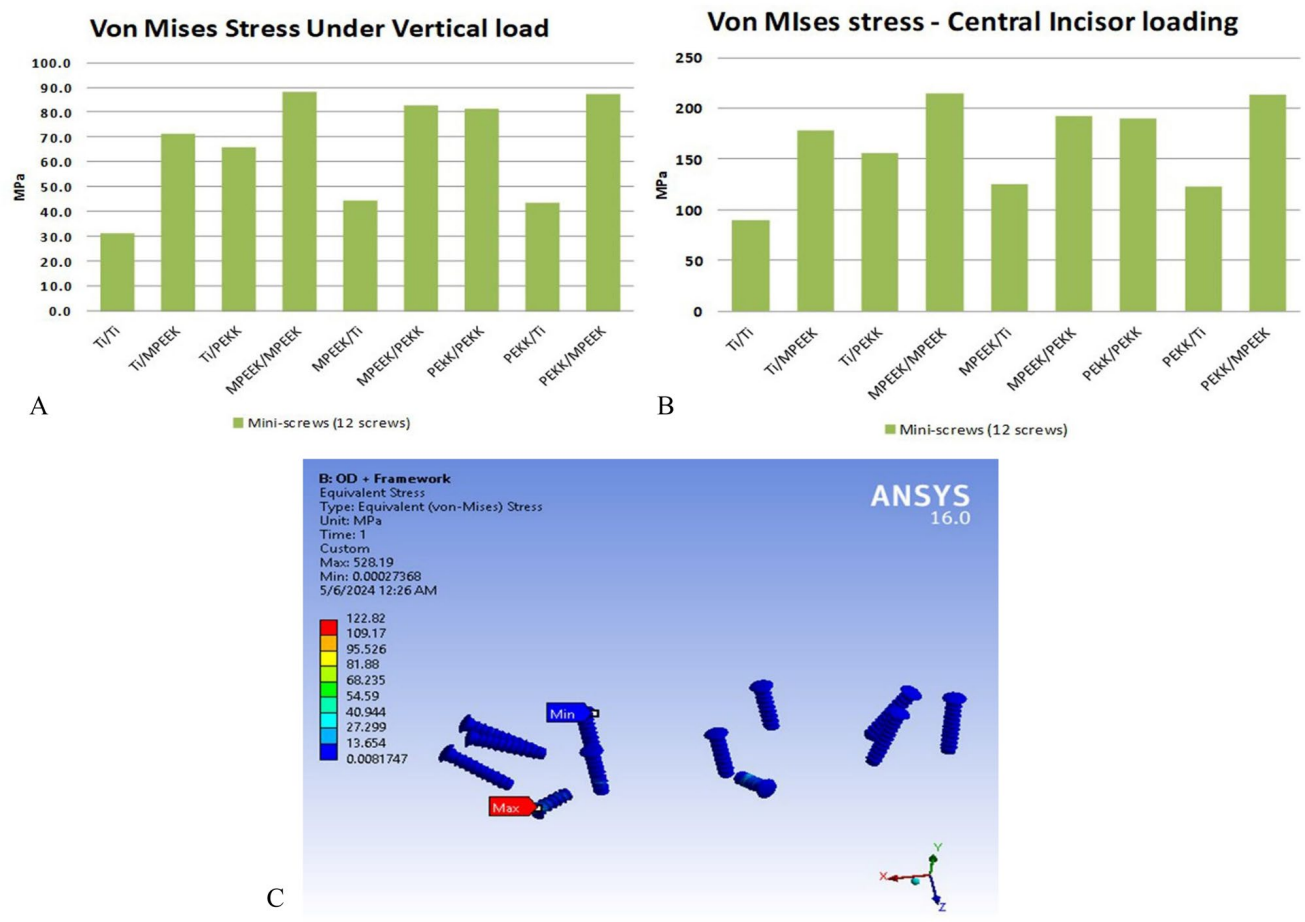
Comparison of stresses extreme values appeared on mini-screws and sample of its stress’s distributions under incisors vertical loading

Mini-screw stress varied by loading and subperiosteal material. Under vertical loading, stresses ranged from 30 MPa (titanium) to 50 MPa (modified PEEK). Oblique loading produced the lowest values (~ 20 MPa with titanium). Under incisor loading, screw stress reached a maximum of 200 MPa (modified PEEK), 190 MPa (PEKK), and 130 MPa (titanium)—all within safe limits for titanium screws (Fig. 9).

Although the primary aim of the FEA was to assess stress and deformation patterns, the results also allowed inference of potential failure modes by comparing stress magnitudes to material strength and fatigue data. The critical stress values refer to those approaching 50–60% of the yield strength in PEKK and modified PEEK—levels associated with fatigue risk based on reported S–N behavior. However, no failure is expected based on S–N curves, as all stresses remained below the endurance limit. Stress peaks at the cement interface under vertical incisor loading may indicate debonding risk. While explicit fatigue simulations were not performed, the findings identify vulnerable regions under anterior loading, particularly for polymer-based frameworks.

**Fig. 10:**
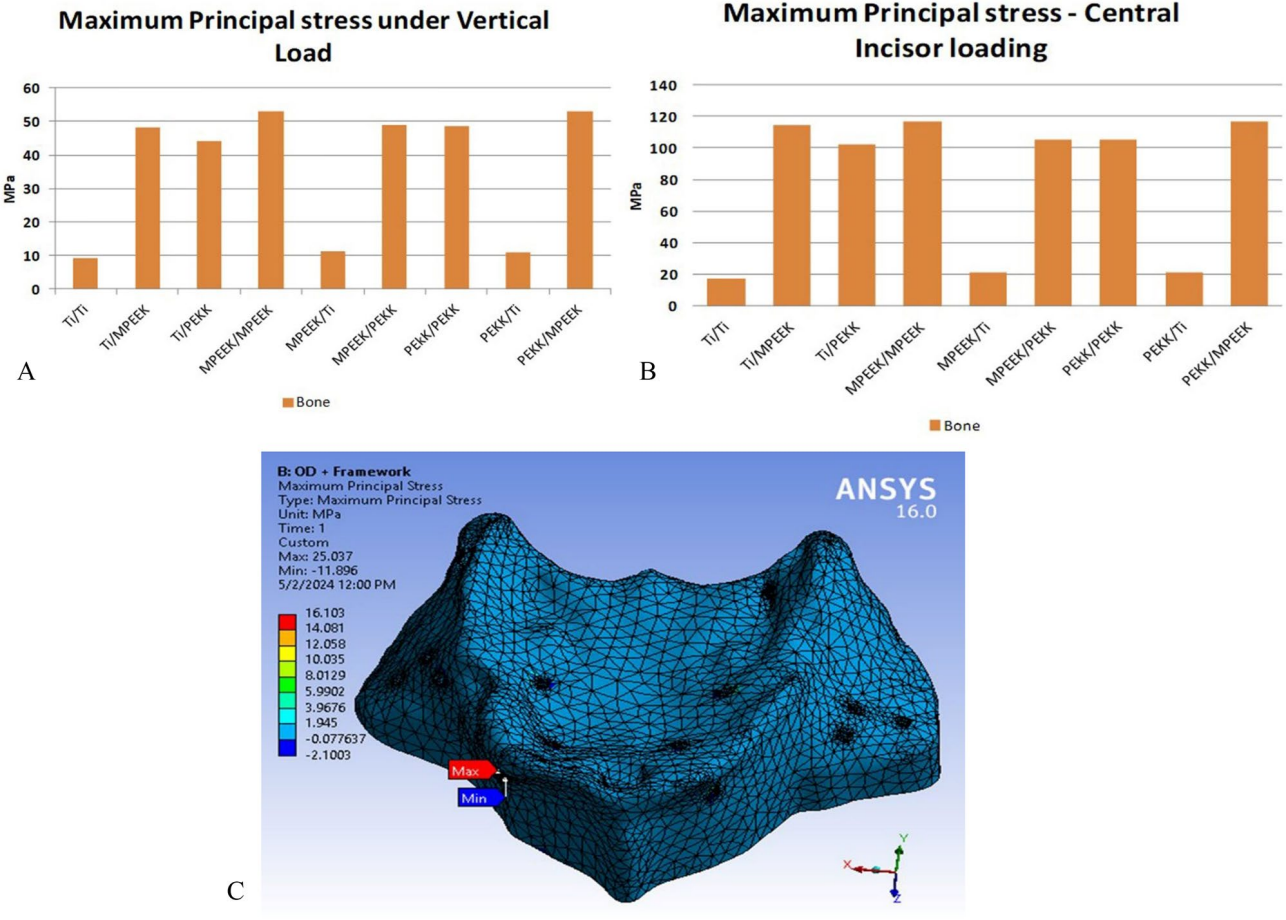
Comparison of stresses: extreme values appeared on bone and a sample of its maximum principal stress distribution for PEKK/modified PEEK frames under oblique loading. PEKK, polyetherketoneketone. PEEK, polyetheretherketone

In all protocols, titanium consistently exhibited superior mechanical behavior, minimizing stress transfer to the bone and screws and maintaining structural stability under loading. Deformation values were lowest for titanium in all cases, followed by PEKK and modified PEEK (Fig. 10). Table 3 outlines how each component (bone, screw, cement, framework) performed under each material combination.

**Table 3:** Performance of (bone, mini-screws, cement, superstructure framework and subperiosteal framework) under different material combinations

Component	Titanium Frameworks	Modified PEEK Frameworks	PEKK Frameworks
Bone	Lowest stress in all loading; safe under all conditions	Highest stress, especially under incisor load; risk of short-term failure	Moderate stress; incisor loading close to yield threshold
Mini-screws	Lowest stress across all loads; within safe limits	Highest under incisor load (~ 200 MPa); within safe limit but near fatigue	High under incisor load (~ 190 MPa); within safe range
Cement Layer	Lowest stress under vertical/oblique load; elevated (~ 35 MPa) under incisor	Highest stress (~ 50 MPa) under incisor; critical range	Moderate (~ 45 MPa); risk of debonding under incisor load
Superstructure Framework	Highest internal stress due to rigidity (up to 160 MPa); but best support to underlying structures	Lowest framework stress (~ 40 MPa); but transferred more stress to bone/screws	Intermediate (45–50 MPa); better balance between flexibility and support
Subperiosteal Framework	Internal stress up to 110 MPa under vertical/oblique; safe and durable	Lower internal stress (~ 55 MPa) but limited fatigue resistance under incisor loading	Moderate (~ 60 MPa); possible short-term fatigue under anterior loads

## Discussion

As there was significant difference in response to stress applied on the bone, superstructure, and titanium screws, the null hypothesis was rejected. This study demonstrates several methodological strengths that enhance its reliability and clinical relevance. Firstly, the use of CT-derived anatomical modeling ensures high anatomical accuracy, closely replicating patient-specific maxillary geometry. Secondly, a detailed finite element mesh design was implemented to optimize result precision, particularly in stress-concentrated regions. Thirdly, the analysis incorporates multiple framework material combinations, including modified PEEK and PEKK, enabling a comprehensive comparison of biomechanical performance. Finally, the application of 3 distinct and clinically relevant loading protocols—including vertical and oblique forces—allows for a nuanced understanding of material behavior under varied functional conditions. These features collectively contribute to the robustness and translational potential of the study’s findings.

Nowadays, zirconia, titanium, and metal alloys are the most often utilized materials in the production of implant-supported prostheses. It has been determined that high-performance polymer materials can enhance framework characteristics and perhaps lower prosthesis costs. Nevertheless, there is a paucity of research on various subperiosteal framework materials. As far as the readers are aware, this is the first FEA study to assess several subperiosteal implant material combinations with various superstructures.

According to studies, the posterior region’s occlusal masticatory forces are approximately 200 N [36]. Since the modeling done here was an elastic analysis, a load of 200 N was selected for the current study. If larger loads were applied, the maximum value should scale directly with the increasing load. In the present study, deformation exerted on all components during analyses showed values within physiological limits of each component, thus there was no possibility of failure of any component under the applied loads which is in agreement with De Moor et al., [20] and Altıparmak et al. [26]

Stresses on the superstructure (teeth and gingival) showed insensitive behavior to exchanging frameworks materials, while the average value of the stress increased according to loading protocol. Generally, no cracks or failures be expected underloading protocols which is in agreement with Attia et al. [37]

Superstructure framework stress values altered according to frameworks materials combination and applied load protocol. In the present study, using titanium as superstructure framework material increases the superstructure framework body stresses dramatically. This increase might refer to titanium rigidity which offers better support to superstructure, and better distribution for the applied loads to underneath structures [38]. Modified PEEK, then PEKK as superstructure framework absorbs the applied load energy better than titanium one, thus, they transfer less load to underneath structures but not well distributed. These results agree with various studies [39–41].

Under incisors vertical loading, the von Mises stress appeared on cement layer reached critical values of order 50 MPa, which indicate short lifetime for the cement layer (debonding of the superstructure). This might be referred to high bending at the anterior support, where the loading site is totally away from supporting points.

On the other hand, under the bilateral vertical and oblique loading, the applied loads located in between the supports which reduces the cement stresses to reasonable values which ensures long lifetime by decreasing the chance of debonding the superstructure. These results are in agreement with Alvarenz-Arenal et al., [42] as the occlusal loading application site influences the bone stress around the implant. Regardless of the location of the implants, unilateral loading significantly increases the peri-implant bone stress on the load side, whereas bilateral occlusal loading distributes it symmetrically.

Subperiosteal frame received high stresses under incisors vertical load, due to high bending, reached 60 to 70 MPa. These values are critical (very short lifetime) for modified PEEK, and PEKK. On the other hand, under bilateral vertical or oblique loading the stresses values were safe for the three tested subperiosteal frame materials which in agreement with Bhering et al., [43] that stiffer framework materials demonstrated the greatest biomechanical behaviour.

Incisors vertical loading exerts the highest level of stress on mini screws. However, these values were safe (no expected failure). The present study demonstrates that titanium subperiosteal frame transferred less stress to screws, followed by PEKK, which may be due to material rigidity.

Bone was safe in all cases except incisors loading cases with subperiosteal frame materials modified PEEK and PEKK, where the maximum tensile stress indicates values close to bone yielding (failure in short term). The trend of titanium subperiosteal frame transferred less stress to bone “underneath structures”, followed by PEKK, then modified PEEK. This trend might be referred to materials rigidity.

In the present study, the term “critical stress” refers to stress levels that approach or exceed the reported yield strength or fall within the high-slope region of the S-N fatigue curve for a given material. For instance, modified PEEK (BioHPP) has a reported yield strength in the range of 110–120 MPa, while PEKK exhibits yield strengths up to 140 MPa depending on processing methods.^2344^ Titanium (Ti-6Al-4 V), in contrast, offers a much higher yield strength of approximately 880–950 MPa [20].

The peak von Mises stresses observed in the subperiosteal frame under incisor vertical loading ranged between 60 and 70 MPa, which, although below the yield strength of PEKK and PEEK, are concerning from a fatigue standpoint. Literature suggests that repeated cyclic loading at 50–60% of the yield strength can lead to failure in **~** 10,000 cycles for polymer-based materials [45] equivalent to roughly one year of typical masticatory function. Hence, “short-term failure” in this context denotes a projected functional lifespan of ≤ 1 year, especially under anterior high-bending loads without occlusal support.

By contrast, titanium’s much higher endurance limit ensures that the same stress levels remain well below both static and fatigue thresholds, implying longer structural survival under identical conditions.

The result of the present study could be applicable in clinical situation as demonstrated by El-Sawy et al., [15] where the maxillary modified PEEK subperiosteal implant opposed by conventional complete denture and no fracture occurs to the framework for more than 2 years follow-up. Similar outcomes may also occur if a PEEK subperiosteal implant is placed solely in the anterior section as opposed to the natural dentition, as the anterior region’s masticatory force is lower than the posterior segment’s [46].

Stress shielding or stress protection occurs when metal implants, screws, or bone plates are used for replacement surgery. Although rigid metallic parts stabilize the prosthesis “better than polymeric ones”, and may allow early loading, the higher stiffness of the implant results in bone loss as a result of decreased physiologic loading of the bone. The modified PEEK and PEKK are similar in properties to bone. Both materials generally offer a Young’s modulus that is significantly lower than titanium, making them potentially better at reducing stress shielding. This advantage of replacing natural tissue with parts having similar properties will be extremely effective with replacing the same shape and dimensions. Unfortunately, this is not applicable in this study, and the loading transfer mechanism is different than natural ones. Therefore, stronger material like titanium has succeeded in achieving the desired stability with acceptable stress in bone within physiological limits.

### Study limitations

This study presents several limitations that should be acknowledged. The materials were modeled as isotropic, homogeneous, and linearly elastic, which simplifies computation but does not fully reflect the anisotropic or time-dependent behavior of bone and polymeric materials such as PEEK and PEKK. Additionally, only static loading conditions were applied, without accounting for fatigue, creep, or long-term degradation—factors that are particularly important for polymers exposed to cyclic forces and the variable environment of the oral cavity. While stress values were compared against known material thresholds to estimate short-term performance, this approach cannot replace dynamic or fatigue testing. The model was constructed using the anatomy of a single patient, which limits the generalizability of the results to broader populations with varying anatomical characteristics and bone densities. Moreover, the lack of experimental validation means that, despite alignment with previous literature, the findings remain theoretical. Finally, the study did not explore clinical translation challenges such as fabrication constraints of polymer frameworks, the long-term reliability of cement-polymer bonding, or the influence of patient-specific variables like bite force variability and parafunctional habits, all of which could significantly impact long-term outcomes.

### Recommendation

Future research should incorporate anisotropic and viscoelastic properties, especially for polymer-based components, to achieve more physiologically accurate simulations. Long-term performance must be assessed through fatigue, creep, and accelerated aging tests. Broader anatomical modeling—via patient-specific data or statistical shape analysis—can enhance result generalizability. In vitro validation and biocompatibility testing, particularly for newer polymers, are also crucial for clinical translation. In addition, assessing how different subperiosteal frameworks influence tissue response and bacterial colonization. Emerging evidence suggests that polymer-based materials may offer superior biocompatibility compared to titanium [47], which could have meaningful clinical implications. Future studies should also explore how the interplay between biomechanical performance and biological integration affects long-term implant success. Investigating these dynamics under realistic, patient-specific conditions will be vital for advancing personalized treatment strategies and improving prosthetic outcomes.

The results of the present study offer a practical reference for selecting framework materials in different clinical scenarios in atrophied maxillae, indicating that titanium remains the material of choice for patients with high masticatory forces and reduced bone quality, or posterior occlusion due to its superior strength, structural rigidity, and long-term fatigue resistance. It is especially suitable for full-arch restorations or cases involving opposing natural dentition or implant-supported prostheses. PEKK and modified PEEK (BioHPP) may serve as viable alternatives in lower-load or more conservative clinical scenarios where reduced stiffness is beneficial for minimizing stress shielding. These materials are appropriate for patients with moderate to low occlusal forces—such as those with opposing complete dentures, geriatric patients with diminished bite force, or anterior-only restorations. While enhanced esthetics and flexibility are advantageous in select cases, their increased susceptibility to fatigue under high-stress conditions should be carefully considered during treatment planning.

## Conclusions

Based on the findings of this FEA analysis, the following conclusion was drawn:

1- Deformation observed in all components during the analysis remains within the physiological limits for each structure.

2- Modified PEEK, then PEKK as superstructure framework absorbs the applied load energy better than titanium one, thus, they transfer less load to underneath structures but not well distributed as titanium one.

3- Titanium subperiosteal frame transfers less stress to underneath structures, followed by PEKK, then modified PEEK, that may refer to materials rigidity. PEKK, then modified PEEK as subperiosteal frame materials may lead to short term failure in cement, subperiosteal frame (of order one year).

## Data Availability

The datasets used in the current study are available from the corresponding author upon request.
